# Influence of Liver Condition and Copper on Selective Parameters of Post-Mortem Dog Tissue Samples

**DOI:** 10.3390/ani8120237

**Published:** 2018-12-13

**Authors:** Isabella Corsato Alvarenga, Charles Gregory Aldrich, Dennis E. Jewell

**Affiliations:** 1 Department of Grain Science & Industry, Kansas State University, Manhattan, KS 66506, USA; isacorsato@ksu.edu; 2 Nutrition Innovation, Hill’s Pet Nutrition, Topeka, KS 66601, USA; dennis_jewell@hillspet.com

**Keywords:** dog, liver, copper, histopathology, cholestasis, oxidation

## Abstract

**Simple Summary:**

The liver is a vital organ involved in numerous physiological functions. Maintaining its health is vital to the wellbeing of the dog. Copper is a transition metal that can cause cell oxidation when stored in excess. This surplus storage in the liver may happen due to breed genetics, or excess dietary copper consumption. The objective of this work was to determine relationship between hepatic copper and liver pathology severity with plasma metabolites, complete blood count, and blood chemistry. Copper accumulation was not related to either liver pathological condition nor to an increase in liver biomarkers in the selected dog population. However, an increasing copper concentration suggested oxidation and cell membrane stress. Liver pathology severity was related to increased liver enzymes, and some cholestasis. Further, liver neoplasia was correlated with biomarkers that suggest rapid cell division and increased energy metabolism.

**Abstract:**

One of the liver functions is copper storage, which can be toxic when in excess. The objective of this retrospective study was to determine the relationship between hepatic copper and pathology conditions in stored samples from 55 post-mortem dogs (37 Beagles, 12 Labrador Retrievers, and 6 Labrador Mixes). The analyses evaluated data from blood chemistry and complete blood count (CBC) that were measured immediately before euthanasia, and liver biopsies which were harvested at necropsy and frozen at −80 °C. Slides for microscopic evaluation were prepared, and liver copper and plasma metabolites were measured. Hepatic copper was correlated (*p* ≤ 0.001) with monoacylglycerols, 13-HODE + 9-HODE (13-hydroxy-9,11-octadecadienoic acid + 9-hydroxy-10,12-octadecadienoic acid), and stearoyl-arachidonoyl-glycerophosphocholine. This indicates lipid metabolism modification and cell membrane oxidation. However, hepatic copper was not related to liver histopathology severity or altered liver biomarkers. The severity of liver pathology was positively correlated (*p* ≤ 0.05) with liver enzymes, bile salts, and glycerophosphocholines, suggesting cholestasis and altered lipid and amino acid metabolism. Liver neoplasia had increased (*p* ≤ 0.05) metabolites derived from nucleotides, along with an increase (*p* ≤ 0.05) in α-ketoglutarate from the energy and amino acid metabolism (*p* ≤ 0.05), suggesting rapid cell division. This study offers further insight regarding changes in metabolism due to hepatic tissue damage.

## 1. Introduction

The liver is a vital organ with multiple physiological functions related to storage, in addition to homeostasis of metabolic, endocrine, and biliary systems. These include important processes such as synthesis and storage of glycogen and triglycerides, gluconeogenesis, synthesis of plasma albumin, fibrinogen, and coagulation factors, synthesis of ammonia from urea, synthesis of bile salts from cholesterol, detoxification of substances before excretion via the bile, and activation of vitamin D, among others [1]. Because of all these functions, pathological alterations in metabolism can lead to liver damage. These are not yet fully understood.

Primary hepatitis in the dog has been reported to be caused by microorganisms, toxins, drugs, and immune-mediated and breed-related metabolic errors [2]. A study reported that the prevalence of dogs with primary hepatic disease was only 0.5% in their first visit to the clinic [2]. Most of these had chronic liver disease and 28.7% were associated with excess copper storage.

Copper is an essential micronutrient in the dog. It is a component of several enzyme systems and plays a role in cellular respiration, hair pigmentation, and bone formation, among others [3]. The liver is responsible for copper metabolism, redistribution within the body, storage, and excretion through the bile [4]. However, when stored in excess, copper can impart oxidative injury through the formation of highly reactive oxygen species, such as hydroxyl radicals [5]. There are three different scenarios that can lead to copper toxicosis: (1) a primary genetic condition, (2) inefficient copper excretion by the bile secondary to liver disease, and (3) excess dietary copper intake [6]. Copper-induced hepatitis associated with mutations in copper metabolism enzymes has been described in Bedlington Terriers, West Highland and Skye Terriers, Doberman Pinschers, Labrador Retrievers, Dalmatians, and Anatolian Shepherd Dogs [2,7]. Dogs with genetic and or secondary copper storage disease rely on dietary restriction for the success of clinical therapy [8]. However, unless unbalanced, diet alone will not cause copper toxicosis in animals without copper storage disease.

The goal of this work was to understand how hepatic copper accumulation and (or) liver pathology condition affected metabolism. Our first hypothesis was that liver copper would be positively related to liver condition severity, to changes in liver enzymes, and (or) oxidation biomarkers. The second hypothesis was that liver condition would impact liver biomarkers and different plasma metabolites.

## 2. Materials and Methods

### 2.1. Animals

All liver samples were from previously deceased dogs in which tissues and medical histories were retrieved from either the Hill’s bioarchive (internal) or from external private Veterinary clinics (from Washington D.C., Kansas, and Texas (USA) and Guelph (Canada)). Samples were from 55 dogs which had been euthanized by a veterinarian when quality of life had severely deteriorated and prior to planning this evaluation. All of the dog owners gave their informed consent for inclusion of their animals’ tissues before participation in the study and the work was conducted in accordance with the Declaration of Helsinki. The protocol was approved by the Institutional Animal Care and Use Committee (IACUC) at Hill’s Pet Nutrition, Inc. (#677.0.0.0). Dogs for the “normal” liver pathology group were selected from a population of banked samples which had not previously been identified with a liver pathology condition. Prospectively, this could have been secondary to another pathology, but was not evident upon initial screening. The “liver pathology” groups of dogs were identified from their ante-mortem records. These samples were separated into liver condition groups according to histopathology results. In total there were 37 Beagles, 12 Labrador Retrievers, and 6 Labrador Mixes. From these, 2 were intact females, 29 were spayed females, and 24 were neutered males. Average age at death was 12.8 years (±3.23; range 0.4–16.4). Demographic information is summarized in Table 1. 

### 2.2. Sample Analysis

Blood was collected a few minutes before the administration of an euthanasia solution (Euthasol, Virbac AH, Inc., Fort Worth, TX, USA). Blood chemistry and CBC were measured within 30 min of collection. Liver biopsies were harvested at necropsy to fit into 1.8-mL cryovials. These were immediately flash frozen in liquid nitrogen and stored at −80 °C until analysis. Microscope slides of each liver sample were stained with hematoxylin and eosin for histopathology evaluation. Plasma metabolites were measured by a commercial laboratory (Metabolon; Durham, NC, USA). Copper concentration in the liver was determined by inductively coupled plasma–optical emission spectrometry (ICP-OES; Optima 4300 DV, PerkinElmer instruments, Waltham, MA, USA). Metabolites in plasma were measured according to the procedure by Evans et al. (2014) [9].

### 2.3. Liver Pathology Classification

Liver samples were assessed microscopically by a Veterinary pathologist either at Hill’s Pet Nutrition or the Kansas State University Veterinary Diagnostic Laboratory (Manhattan, KS, USA). Dogs were divided into five groups according to liver histopathology findings: normal, mild, moderate, severe, and liver neoplasia. Liver histopathology was classified based on a 10-point scale, where 3–4 was mild, 5–6 was moderate, and 9–10 was severe. Mild liver condition was characterized by a small amount of change, which included, but was not limited to, inflammatory cell infiltration, vacuolar degeneration, pigment accumulation, and hyperplasia. Moderate liver histopathology was classified by a more significant amount of change, which included, but was not limited to, inflammatory cell infiltration, vacuolar degeneration, pigment accumulation, and hyperplasia, with some disruption of normal architecture. Finally, severe liver conditions were those that had a very significant amount of change, which included, but were not limited to, inflammatory cell infiltration, vacuolar degeneration, pigment accumulation, and hyperplasia, with significant disruption of normal architecture. The extent of liver neoplasia could not be captured because the study was limited to using slides of previously collected dog liver samples. Thus, there was no gross pathology evaluation of each subject.

There were 16 dogs within the normal group. The primary clinical diagnoses of the normal group included arthritis (1), cardiac disease (1), enteritis (1), foreign body (1), neoplasia (2), neurologic dysfunction (2), renal disease (4), and unknown causes of death (4). There was not a true control in this study because all samples were from previously deceased dogs which had some type of terminal health condition. Nineteen liver samples were classified as mild, and histopathology findings included mild inflammation, nodular and/or biliary hyperplasia, vacuolar degeneration, fibrosis, and pigmentation (hemosiderin). Primary clinical diagnoses of the mild group included arthritis (2), cardiac disease (1), chronic diarrhea and weight loss (1), chronic pain (1), Cushing’s disease (1), dementia (1), laryngeal paralysis (1), neoplasia (5), renal disease (3), and unknown (3).

Liver histopathology of 9 dogs were classified as moderate, and findings included moderate necrosis, congestion, cholestasis, vacuolar degeneration, hemorrhage, and nodular and/or biliary hyperplasia. In this group, 4 dogs had liver disease as primary cause of death, 3 had neoplasia, and 3 were unknown. Dogs with severe liver histopathology findings (6) had at least one of each characteristic: marked vacuolar degeneration, and nodular and/or biliary hyperplasia. In the severe group there were 3 cases of liver disease and 3 different primary clinical diagnoses (abscessed lipoma, renal disease and seizures). Finally, liver neoplasia was considered as a separate group due to its peculiarities. The types of neoplasia in this group were lymphosarcoma (2), metastatic hemangiosarcoma (1), metastatic adrenal cortical carcinoma (1), and a metastatic pheochromocytoma (1).

### 2.4. Statistical Analysis

Statistical analysis of liver copper concentration on a dry weight basis related to plasma metabolites were performed as a bivariate analysis using statistical software (JMP v 12.1.0, Copyright © 2015 SAS institute Inc., Cary, NC, USA). Liver copper concentrations were natural log transformed before statistical analysis in an attempt to normalize the distribution. Hepatic copper was the fixed continuous variable, while plasma metabolites were considered the continuous dependent variables. Pearson correlations were considered significant at *p* ≤ 0.001.

The experiment was conducted as a complete randomized design (CRD). Mean plasma metabolites from groups of dogs classified according to liver pathological condition at time of death (normal, *n* = 16; mild, *n* = 19; moderate, *n* = 9; severe, *n* = 6; neoplasia, *n* = 5) were separated by analysis of variance (ANOVA) with the aid of a statistical software (PROC GLIMMIX, SAS, version 9.4., SAS Institute Inc., Cary, NC, USA), and were considered significant at *p* ≤ 0.05. Bonferroni correction was applied. Aitchison’s centered log ratio transformation [10] was performed on plasma metabolites before the analysis. Blood chemistry and complete blood count (CBC) parameters were natural log transformed before statistical analysis to normalize distributions. Means of blood CBC parameters of dogs grouped by liver pathology condition were separated by ANOVA using statistical software (PROC GLIMMIX, SAS, version 9.4., SAS Institute Inc., Cary, NC, USA). There were two sites of analysis for CBC and blood chemistry (external and internal), so ‘site of analysis’ variable was included in the random statement.

## 3. Results

### 3.1. Copper Correlation with Plasma Metabolites

**Figure 1:**
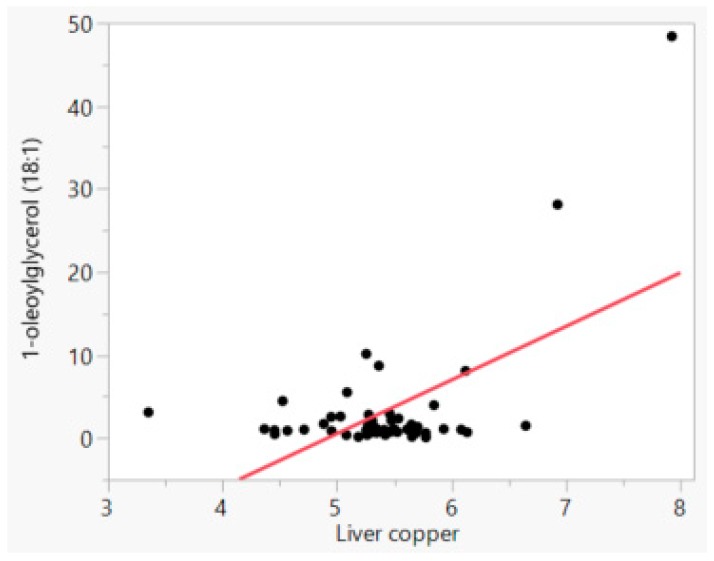
Relationship between natural log liver copper (ppm) and natural log 1-oleoylglycerol (18:1, ppm) concentrations.

In this study there were only two dogs with liver copper levels above 1000 ppm, and they belonged to breeds which are more prone to suffer from oxidative damage. These animals were the main drivers of all the Pearson correlations. An example is illustrated in Figure 1, which shows the highest correlation in the study between copper and a molecule from the lipid metabolism pathway (1-oleoyl-glycerol). Although the observation with highest concentration (2768 ppm or 7.93 ln copper; Figure 1) was a statistical outlier, we decided to keep it in the data due to its important biological significance. Hepatic copper concentration had a positive correlation to a few metabolites from the lipid metabolism pathways. Copper correlated (p ≤ 0.001) with monoacylglycerols 1-oleoylglycerol (18:1), 1-linoleoylglycerol (18:2), 2-linoleoylglycerol (2-monolinolein) and 1-arachidonylglycerol (R = 0.58, 0.56, 0.50 and 0.42, respectively; Table 2). Monohydroxy fatty acid 13-HODE + 9-HODE and stearoyl-arachidonoyl-glycerophosphocholine (2) also correlated to copper (p ≤ 0.001), but to a lesser extent (R = 0.44 and 0.43, respectively).

### 3.2. Plasma Metabolites of Different Liver Pathology Conditions

A total of 523 metabolites were measured in the plasma samples, which were classified as part of amino acid (162), carbohydrate (23), cofactors and vitamins (25), energy (7), lipid (177), nucleotide (34), peptide (23) and xenobiotics (72) metabolism. Out of these analyses, plasma metabolites that had significant differences (*p* ≤ 0.008) among liver pathology groups were reported in Table 3. It was expected that more severe liver conditions would have a greater copper accumulation in hepatocytes, but copper concentration was not different among groups (*p* > 0.05; Table 3).

Compounds from the nucleotide metabolites 1-methyladenosine, xanthosine and N-acetyl-beta-alanine were greatest (*p* ≤ 0.05; Table 3) in the neoplasia group (1.84, 5.48 and 2.50 vs. averages 1.03, 1.04, and 1.08, respectively). Alpha-ketoglutarate from energy metabolism was also highest (*p* ≤ 0.05) in neoplasia compared to normal, mild and moderate groups (3.58 vs. average 1.05), but the severe group was similar to the extremes (1.325). Alpha-ketobutyrate from amino acid catabolism was greater (*p* ≤ 0.05) in the neoplasia group compared to normal, mild and severe groups (3.06 vs. average 1.09), and the moderate group was similar to the extremes (1.54). The amino acid asparagine was greater (*p* ≤ 0.05) in the severe compared to normal, mild and moderate groups (2.11 vs. average 1.03), with neoplasia similar to the extremes (1.28). Glutamate was greater (*p* ≤ 0.05) in the neoplasia than the normal group (2.812 vs. 0.903), and mild, moderate and severe groups were similar to all.

The majority of metabolites with significant differences among groups were from lipid metabolism. The 2-aminoheptanoate concentration was almost 5-fold higher (*p* ≤ 0.05) in the severe than normal, mild and moderate groups, with results for neoplasia group similar to the extremes (2.29). The severe group had a 5-fold increase (*p* ≤ 0.05) in plasma taurolithocholate compared to the average of all other groups. Plasma taurocholate of the severe group was 14-fold greater than the average of normal and mild groups (16.47 vs. average 1.185), and moderate and neoplasia groups were similar to the extremes. The 1-arachidonoyl-GPC (20:4) concentration was also related to severity; wherein, plasma of normal, mild and neoplasia groups were the lowest (*p* ≤ 0.05; average 0.93) and the severe group had the highest (2.74) concentration, with the moderate group similar to all others (1.36). Concentration of 1-oleoyl-GPC (18:1) and 2-palmitoyl-GPC (16:0) were both higher (*p* ≤ 0.05) in the severe group (2.53 and 2.92, respectively) than normal and mild groups (averages 0.99 for 1-oleoyl and 1.06 for 2-palmitoyl), but not different from the moderate and neoplasia groups (averages 1.41 for 1-oleoyl and 1.31 for 2-palmitoyl).

### 3.3. Blood Chemistry and Complete Cell Count of Different Liver Conditions

Blood chemistry parameters used in the statistical analysis included glucose, blood urea nitrogen (BUN), creatinine, total protein, albumin, total bilirubin, alkaline phosphatase (ALP), alanine aminotransferase (ALT), cholesterol, calcium, phosphorous, sodium, potassium, chloride, albumin:globulin, BUN:creatinine, globulin, and triglycerides. Complete blood count parameters used in the statistical analysis included hemoglobin, hematocrit, white blood cells (WBC), red blood cells (RBCs), mean corpuscular volume (MCV), mean corpuscular hemoglobin (MCH), mean corpuscular hemoglobin concentration (MCHC), and platelet count. No blood chemistry or CBC parameters had a significant relationship with copper concentration in the liver (*p* > 0.05; data not shown), but some were different among groups of dogs separated by liver condition. Liver enzymes ALT and ALP increased according to severity, as expected: Natural log of ALT was higher (*p* ≤ 0.05) in the moderate and severe groups than in the normal group (average 5.20 vs. 3.38), and mild and neoplasia were similar to all others (Table 4). Alkaline phosphatase (ALP) was greater in the severe than the normal group (7.04 vs. 5.11), and mild, moderate and neoplasia groups were similar to the extremes (average 5.77). Calcium was lowest (*p* ≤ 0.05) in the neoplasia group compared to normal and mild groups (2.16 vs. average 2.33), and moderate and severe groups were similar to extremes. Platelet count was lowest (*p* ≤ 0.05) in the neoplasia group compared to all others (5.14 vs. average 6.11). Finally, globulin was greater (*p* ≤ 0.05) in moderate than neoplasia groups (1.23 vs. 0.836), and the other groups were similar to both.

## 4. Discussion

### 4.1. Effect of Copper Concentration on Plasma Metabolites

This study was undertaken with harvested tissues from dogs that had been euthanized due to various terminal conditions. These tissues were banked at that time, along with collection and analyses of blood and plasma samples. While addressing liver copper and metabolic diseases may not be ideal under these circumstances as the underlying conditions leading to their euthanasia may have also influenced liver and whole-body health, that some clues about conditions leading to the disease state are no less meaningful. The intent of this work is not to create a definitive causality, but rather provide some insight into the pathology that future work might reveal in a more direct path. 

Samples from Labrador Retrievers and Labrador Mixes were selected for the present study because there are reports of inherited copper-associated hepatitis [4,6], and they may be the most prevalent breed with liver disease [2]. In our study, samples from 5 out of 6 dogs with increased liver copper measured by ICP-OES were Labradors. Beagle dog samples were selected either to be part of the normal liver condition group or because they presented primary hepatic disease diagnosis.

From the dog samples selected for this study, only 6 had copper concentrations above 400 ppm on a dry weight basis, which is considered above the upper limit according to Fieten et al., 2014 [4]. However, dog samples with highest hepatic copper concentrations (1020 and 2768 ppm) did not have abnormal liver pathology, and the other animals with increased copper were included in the mild and moderate groups. This happened because copper was measured by ICP-OES instead of being visualized in the slides under the microscope as others have previously reported. For example, in a retrospective study by Poldervaart et al. [2] the authors reported that 24 of 101 dogs with liver disease in their population died from chronic hepatitis associated with copper storage. They determined copper accumulation using a staining technique to grade and stratify dogs to condition rather than the actual measurement of copper via ICP-OES used in our work. 

Interestingly, copper concentration was also not related to any of the blood chemistry or CBC parameters, including most well-known liver markers. Johnston et al. (2013) [12] also reported in a retrospective study that many Labrador Retrievers with high liver copper concentration, ranging from 93 to 3810 ppm (average 299 ppm), did not present with copper related toxicosis or any reported liver disease. In their study they reported that the average liver copper concentration in asymptomatic dogs had increased since 2000 when pet food companies were no longer allowed to use cupric oxide in their formulas and replaced it with more bioavailable copper forms [13].

Although copper did not correlate with any well-known liver marker, it did correlate with plasma metabolites that belong to the lipid metabolic pathways, such as monoacylglycerols. The liver is a key organ in energy metabolism and fat oxidation, so it would be expected that some types of lipids would be altered when hepatic tissue is damaged. Diacyl- and triacylglycerides can be synthetized either from glycerol-3-phosphate through the glycerol phosphate pathway or from monoacylglycerols through the monoacylglycerol acyltransferase pathway [14]. Dogs with high hepatic copper in this study had an increase in 4 different monoacylglycerols, but no increase in diacyl- and triacylglycerides. A possible explanation is a malfunctioning of the monoacylglycerol acyltransferase, causing monoacylglycerols to accumulate in the liver and leak into the blood stream.

There was also an increase in oxidative markers in dogs with high copper. The metabolites 13-HODE + 9-HODE are products of linoleic acid oxidation, which is a common process in mammals after tissue injury [15]. The increase of this lipid peroxidation product in animals with high copper accumulation indicates cell death, which can be caused by transition metals. In mice, elevated copper was shown to accumulate in the cytosol of hepatocytes and to decrease serum oxidase activity [16]. In the present study the activity of this enzyme was not measured, but there was an indication of oxidation in some dogs. It is also important to emphasize that the reported causes of death of the two dogs with highest copper (main drivers of the correlations) were neurologic disease and squamous cell carcinoma. These disease conditions may also lead to oxidative damage and could have a large role in the oxidation metabolites.

Finally, stearoyl-arachidonoyl-glycerophosphocholine is a part of the phosphatidylcholine metabolism [17]. Phosphatidylcholines are a major component of mammalian cells and it is critical for maintaining homeostasis of membrane structure and function [18]. Thus, the increase in plasma stearoyl-arachidonoyl-glycerophosphocholine could be an indication of cell membrane disruption caused by oxidation induced by copper and (or) the disease states of these dogs.

### 4.2. Effect of Liver Condition on Plasma Metabolites

Neoplasia is a pathological condition where abnormal cell proliferation occurs and metabolism deregulates. A study from 1980 reported that samples of liver hepatomas in mice had increased amounts of nucleotides, which were due to an increased rate of cell division [19]. The same phenomenon was observed in the present study, wherein precursors of nucleotides 1-methyladenosine, xanthosine and *N*-acetyl-beta-alanine were greatest in dogs with liver neoplasia. Energy metabolism was altered in both the neoplasia and severe groups due to an increase in plasma α-ketoglutarate. This metabolite is a rate-determining intermediate in the tricarboxylic acid cycle (TCA) and is essential for energy metabolism [20]. Alpha-ketoglutarate concentration is controlled by the α-ketoglutarate dehydrogenase complex. This enzyme is a major modulator of the electron transport chain and is also pivotal to the energetic requirements of cancer cells [21]. The accumulation of NADH in cancer cells may inhibit NADH-dehydrogenase, thus decreasing α-ketoglutarate oxidation [21]. This could lead to α-ketoglutarate build-up and leakage into plasma.

Three metabolites from the amino acid metabolism were altered in both severe and neoplasia groups. Alpha-ketobutyrate plays a role in cysteine synthesis from methionine, and it is also a gluconeogenic substrate [22]. The increase of this compound in plasma indicates a boost in energy metabolism. Asparagine can be synthesized endogenously from glutamine by asparagine synthetase. Besides being a constituent of proteins, asparagine is a precursor of the glucogenic amino acid L-aspartate, and it also plays a crucial role in ammonia detoxification in the liver [23]. Thus, the elevation of plasma asparagine of dogs with severe liver conditions could mean an increase in energy production with a rise in detoxification rate due to the disease state and tissue damage. Glutamate is an amino acid synthesized from glutamine that was also elevated in the severe group. It is the most abundant intracellular amino acid and has the ability to donate its amino group for new amino acid synthesis [24]. Humans with steatohepatitis were also reported to have elevated glutamate [25]. The α-amino acid 2-aminoheptanoic acid [26] increased in the severe group, which is another indicator of altered amino acid metabolism.

The 5-fold increase in plasma taurolithocholate, and 16-fold increase in plasma taurocholate of the severe group indicate cholestasis. These are conjugated bile salts, which are synthesized from cholesterol and taurine in the liver [27]. The taurine conjugate of lithocholate, taurolithocholate, is part of the monohydroxylated bile salts and it has been suggested to play a role in liver dysfunctions associated with cholestasis [28,29]. A study with humans reported an increase in plasma bile salt precursors in individuals with steatosis and steatohepatitis compared to controls [25]. In the present study some animals likely accumulated taurolithocholate and taurocholate in the liver canalicular system, which leaked out to the blood stream. Taurocholate was extremely high in the severe group due to an individual dog diagnosed with severe liver degeneration.

Lysolipids 1-arachidonoyl-GPC (20:4), 1-oleoyl-GPC (18:1), and 2-palmitoyl-GPC (16:0) also increased according to degree of liver severity. Lysolipids are synthesized by the enzyme phospholipase A2 (PLA2) and constitute 0.5% to 6% of the lipid weight of healthy mammalian cell membranes [30,31]. However, elevated levels of lysolipids can contribute to several inflammatory conditions [30]. In plasma, significant amounts of lysophosphatidylcholine are formed by a specific enzyme system, lecithin:cholesterol acyltransferase (LCAT), which is secreted by the liver [32]. In the present study the increase in plasma lysolipids of dogs with severe liver disease could have been caused by a possible increase in LCAT activity, with concomitant elevation of inflammatory compounds.

### 4.3. Effect of Liver Condition on Blood Chemistry and Cell Blood Count

Biochemical analysis of serum with increase in liver enzymes alanine aminotransferase (ALT) and alkaline phosphatase (ALP) are usually the first indicators of hepatic disease. Marked elevation of ALT suggests hepatocyte necrosis [33]. Alkaline phosphatase encompasses a group of several enzymes and can be associated with numerous non-hepatic functions, although it is commonly associated with hepatopathy [34]. In the present study both liver enzymes were above the normal limits in the severe group. Liptak et al. (2004) [35] also found that dogs with impaired liver had increase in ALT and ALP enzymes. The mean ALP was elevated for dogs in all pathology groups according to Tvedten [11]. However, both the internal and external clinics had a wider reference range for ALP than the reference used [11], which may affect the interpretation.

Liver conditions were also related to changes in calcium, platelet count and globulin from CBC and blood chemistry analyses. Hypocalcemia is a common laboratory abnormality among critically ill patients [36]. This phenomenon seems to be multifactorial with proposed mechanisms including altered D-vitamin metabolism and (or) the presence of proinflammatory cytokines and calcitonin [36]. Some of these factors could be responsible for the hypocalcemia in the neoplasia group from our study. Although platelet count was within the normal limits for all groups, it was lower in the neoplasia group compared to the others. The liver plays a central role in coagulation, fibrinolysis and in platelet count regulation [37], so an abnormal platelet count would be expected in impaired hepatic tissue. Globulin was also within the normal limits for all groups. Nevertheless, the interpretation of globulin alone is usually not of clinical importance because the decrease in actual albumin/globulin ratio is needed to confirm an acute phase reaction in infection or inflammation in dogs [38,39].

## 5. Conclusions

Unlike what was hypothesized, copper accumulation was not related to either liver pathological condition or an increase in liver biomarkers. However, copper did correlate with some metabolites from the lipid metabolic pathways, including 13-HODE + 9-HODE and stearoyl-arachidonoyl-glycerophosphocholine. This suggests oxidation and cell membrane disruption, as a cause reflecting the impact of stress agents in the liver. When dog samples were grouped according to liver pathology, liver enzymes increased according to condition severity and some blood biochemistry compounds were altered. Further, dogs with liver neoplasia had increased nucleotide, energy, and amino acid metabolism, suggesting rapid cell division, increased metabolism, and energy expenditure, respectively.

## Figures and Tables

**Table 1:** Demographic data summarized by liver pathology condition (normal, mild, moderate, neoplasia, and severe) at the time of death.

Liver Pathology	Normal	Mild	Moderate	Severe	Neoplasia	Total
N	16	19	9	6	5	55
Breed (B/L/LM)	14/2/0	11/5/3	5/2/2	4/1/1	3/2/0	37/12/6
Age average ± SD (range)	12.1 ± 4.47 (0.4–16.4)	12.6 ± 2.82 (4.5–16.3)	13.5 ± 3.02 (8–16.3)	13.8 ± 1.74 (11–15.9)	12.7 ± 2.22 (10.3–15)	12.8 ± 3.23 (0.4–16.4)
Gender (IF/SF/NM)	1/9/6	1/12/6	0/5/4	0/2/4	0/1/4	2/29/24
Liver copper (dw; ±SD, range)	401 ± 667.9 (86.1–2768)	294 ± 142.2 (142–773)	207 ± 128.8 (78.8–456)	212 ± 110.8 (28.7–378)	203 ± 66.0 (92.5–254)	294 ± 375.1 (28.6–2768)

B = Beagle, L = Labrador, LM = Labrador Mix; IF = intact female, SF = spayed female, NM = neutered male, dw = dry weight, SD = standard deviation.

**Table 2:** Significant correlations (*p* ≤ 0.001) of plasma metabolites with natural log liver copper concentration on a dry weight basis.

Plasma Metabolites (*N* = 55)	R	*p*-Value
1-oleoyl glycerol (18:1)	0.581	<0.0001
1-linoleoylglycerol (18:2)	0.557	<0.0001
2-linoleoylglycerol (2-monolinolein)	0.504	<0.0001
1-arachidonylglycerol	0.425	0.0012
13-HODE + 9-HODE	0.436	0.0009
Stearoyl-arachidonoyl-glycerophosphocholine (2)	0.427	0.0011

13-HODE = 13-hydroxy-9,11-octadecadienoic acid; 9-HODE = 9-hydroxy-10,12-octadecadienoic acid.

**Table 3:** Plasma metabolites (mean ± standard error) with significant differences (*p* ≤ 0.05) among liver pathology groups.

Parameter	Normal	Mild	Moderate	Severe	Neoplasia	*p*-Value
*N*	16	19	9	6	5	
Copper ^1^	5.56 ± 0.254	5.63 ± 0.238	5.19 ± 0.284	5.19 ± 0.324	5.29 ± 0.344	0.3314
1-methyladenosine	0.863 ^b^ ± 0.0862	1.124 ^b^ ± 0.0791	0.966 ^b^ ± 0.1149	1.187 ^b^ ± 0.1407	1.845 ^a^ ± 0.1542	<0.0001
Xanthosine	1.52 ^b^ ± 0.559	1.27 ^b^ ± 0.513	0.62 ^b^ ± 0.745	0.76 ^b^ ± 0.913	5.48 ^a^ ± 1.000	0.0035
*N*-acetyl-beta-alanine	1.222 ^b^ ± 0.1921	1.251 ^b^ ± 0.1763	1.062 ^b^ ± 0.2562	0.795 ^b^ ± 0.3138	2.500 ^a^ ± 0.3437	0.0073
α-ketoglutarate	0.946 ^b^ ± 0.3185	0.982 ^b^ ± 0.2923	1.211 ^b^ ± 0.4247	1.325 ^ab^ ± 0.5201	3.581 ^a^ ± 0.5698	0.0030
α-ketobutyrate	1.153 ^b^ ± 0.2736	1.287 ^b^ ± 0.2511	1.543 ^ab^ ± 0.3648	0.837 ^b^ ± 0.4468	3.165 ^a^ ± 0.4895	0.0074
Asparagine	1.07 ^b^ ± 0.1476	1.01 ^b^ ± 0.1355	1.01 ^b^ ± 0.1968	2.11 ^a^ ± 0.2411	1.28 ^ab^ ± 0.2641	0.0035
Glutamate	0.903 ^b^ ± 0.3118	1.097 ^ab^ ± 0.2861	1.167 ^ab^ ± 0.4157	1.921 ^ab^ ± 0.5091	2.812 ^a^ ± 0.5577	0.0373
2-aminoheptanoate	1.26 ^b^ ± 0.719	1.62 ^b^ ± 0.660	1.28 ^b^ ± 0.959	6.78 ^a^ ± 1.175	2.29 ^ab^ ± 1.287	0.0030
Taurolithocholate	0.306 ^b^ ± 0.1711	0.341 ^b^ ± 0.1570	0.277 ^b^ ± 0.2281	1.669 ^a^ ± 0.2794	0.399 ^b^ ± 0.3061	0.0014
Taurocholate	1.469 ^b^ ± 2.4617	0.901 ^b^ ± 2.2590	4.541 ^ab^ ± 3.2822	16.466 ^a^ ± 4.0199	1.789 ^ab^ ± 4.4036	0.0215
1-Arachidonoyl-GPC (20:4)	0.765 ^b^ ± 0.2542	1.134 ^b^ ± 0.2333	1.360 ^ab^ ± 0.3390	2.737 ^a^ ± 0.4151	0.882 ^b^ ± 0.4548	0.0042
1-oleoyl-GPC (18:1)	0.792 ^b^ ± 0.2258	1.191 ^b^ ± 0.2072	1.379 ^ab^ ± 0.30113	2.527 ^a^ ± 0.3688	1.443 ^ab^ ± 0.4040	0.0057
2-palmitoyl-GPC (16:0)	0.936 ^b^ ± 0.2629	1.184 ^ab^ ± 0.2412	1.437 ^ab^ ± 0.3505	2.921 ^a^ ± 0.4293	1.180 ^ab^ ± 0.4702	0.0057

^1^ Copper concentration is expressed as natural log, while other metabolites are expressed as Aitchison’s centered log ratio. ^ab^ Means with unlike superscripts differ. Results expressed as means ± standard error.

**Table 4:** Natural log of parameters from blood chemistry and complete blood count (CBC) that had significant differences (*p* < 0.05) among liver pathology groups.

Parameter	Normal	Mild	Moderate	Severe	Neoplasia	*p*-Value
ALT ^1^ (N)	3.38 ^b^ ± 0.307 (16)	4.22 ^ab^ ± 0.278 (19)	5.00 ^a^ ± 0.377 (9)	5.41 ^a^ ± 0.452 (6)	4.55 ^ab^ ± 0.491 (5)	0.0008
ALP ^2^ (N)	5.11 ^b^ ± 0.307 (16)	5.39 ^ab^ ± 0.282 (19)	5.93 ^ab^ ± 0.410 (9)	7.04 ^a^ ± 0.502 (6)	5.99 ^ab^ ± 0.550 (5)	0.0240
Calcium ^3^ (N)	2.35 ^a^ ± 0.025 (16)	2.32 ^a^ ± 0.024 (18)	2.24 ^ab^ ± 0.034 (9)	2.28 ^ab^ ± 0.042 (6)	2.16 ^b^ ± 0.045 (5)	0.0040
Platelet count ^4^ (N)	6.01 ^a^ ± 0.172 (16)	5.88 ^a^ ± 0.161 (18)	6.22 ^a^ ± 0.216 (7)	6.32 ^a^ ± 0.228 (6)	5.14 ^b^ ± 0.243 (5)	0.0016
Globulin, g/dL ^5^ (N)	1.010 ^ab^ ± 0.1296 (16)	1.046 ^ab^ ± 0.1281 (13)	1.230 ^a^ ± 0.1416 (5)	1.114 ^ab^ ± 0.1466 (6)	0.836 ^b^ ± 0.1466 (5)	0.0260

^ab^ Means with unlike superscripts differ. Results expressed as means ± standard error. ALT = alanine aminotransferase; ALP = alkaline phosphatase. ^1^ Normal range: 10–94 IU/L (Tvedten, 2004) [11]; natural log range: 2.30–4.54. ^2^ Normal range: 0–300 IU/L (Tvedten, 2004) [11]; natural log range: 0–4.50. ^3^ Normal range: 9.0–11.9 mg/dL (Tvedten, 2004) [11]; natural log range: 2.20–2.48. ^4^ Normal range: 164–510 × 10^3^/dL (Tvedten, 2004) [11]; natural log range: 5.10–6.23. ^5^ Normal range: 1.5–3.5 g/dL (Tvedten, 2004) [11]; natural log range: 0.406–1.253.
